# Two trade names of deferasirox (Osveral® and Exjade®) in reduction of iron overload parameters in major beta-thalassemia patients: A randomized open labeled clinical trial

**DOI:** 10.22088/cjim.13.1.61

**Published:** 2022

**Authors:** Mohammadreza Rafati, Hossein Karami, Bita Lashtoo-Aghaee, Bahareh Lashtoo-Aghaee, Mojdeh Dabirian, Razieh Avan

**Affiliations:** 1 Pharmaceutical Research Center, Department of Clinical Pharmacy, Mazandaran University of Medical Sciences, Sari, Iran; 2 Department of Pediatrics, College of Medicine, Mazandaran University of Medical Sciences, Sari, Iran; 3 Emam Sajjad Hospital, Ramsar, Iran; 4 Department of Microbiology, College of Medicine, Tehran University of Medical Sciences, Tehran, Iran; 5 Department of Cardiology, College of Medicine, Mazandaran University of Medical Sciences, Sari, Iran; 6 Department of Clinical Pharmacy, Medical Toxicology and Drug Abuse Research Center (MTDRC), Faculty of Pharmacy, Birjand University of Medical Sciences, Birjand, Iran

**Keywords:** Deferasirox, Osveral, Exjade, Ferritin, MRI T2*

## Abstract

**Background:**

Beta-thalassemia major patients typically require chronic transfusion and iron-chelating agents to reduce serum iron overload. Osveral^®^ is an available Iranian brand name of deferasirox used by majority of thalassemic patients. The aim of this study was to compare the efficacy of Osveral^®^ vs. Exjade^®^ in major beta- thalassemia patients.

**Methods:**

In this randomized clinical trial, all patients received a single daily dose of 30 mg/kg either of Osveral^®^ or Exjade^® ^for 6 months. Primary outcome was the mean of bimonthly changes in serum ferritin concentration and secondary outcomes included mean changes of heart and liver MRI T2* after a year.

**Results:**

Finally, 80 patients completed the study. The mean serum ferritin level at the end of sixth month significantly decreased in Osveral^®^ and Exjade^®^ groups (p<0.01). After a year, means cardiac MRI T2* in Osveral^® ^group were changed from 25.9±9.6 ms to 25.4±9.7 ms and in Exjade^®^ group from 24.8±9.2 ms to 26.9±5.9 ms, with no significant difference (P=0.43). Mean liver MRI T2* for Osveral^®^ and Exjade^®^ groups were 8.6±6.4 ms (baseline 6.3±4.7) and 6.3±4 ms (baseline 4.9±3.5), respectively and there was no significant difference between two study arms (P=0.1).

**Conclusion:**

Osveral^®^ decreased significantly the serum ferritin level and improved heart and liver iron overload as efficient as Exjade^®^. It can be a suitable cost-effective alternative agent in beta-thalassemia major patients.

Beta-thalassemia major is a hereditary hemolytic anemia defined by ineffective erythropoiesis and hemolysis that is treated with multiple blood transfusions (1, 2). Between 300,000 and 400,000 infants are born with a serious hemoglobinopathies each year and up to 90% of these births happen in low- or middle-income countries (3). The total prevalence ranges of beta-thalassemia are nearly from 3 to 100 patients per 100,000 population in different provinces and approximately 25% of the annual blood products are used for patients with thalassemia (4). Individuals with thalassemia major typically require chronic hypertransfusion to treat severe, symptomatic anemia. Iron overload is inevitable; monitoring iron stores and the use of iron chelator are integral components of therapy. The body excretes approximately 1 mg of iron per day while 250 mg of iron is in a unit of transfused red blood cells (5). In thalassemia patients, iron overload is a common complication which can cause organ damage and increase mortality (6, 7). After 1 year regular transfusions by these patients, iron deposited in parenchymal tissues (8).

Serious and irreversible organ injury, including cirrhosis, diabetes, heart disease, and hypogonadism can be induced following excess iron (9). There was directly a correlation between liver fibrosis and age of patient, concentration of liver iron and number of units transfused (10).

Now, there are three iron chelating agents that can be used to decrease the body burden of iron in transfusion-dependent thalassemia patients. Deferoxamine (Desferal^®^) is a safe and effective iron-chelating agent and has been the standard iron chelator in patients needing for long-term transfusion since the 1970s (11). Desferal is not active orally and has a short plasma half-life, so it is used as long parenteral infusions. Usually, it is given as an overnight subcutaneous by a portable infusion pump for 5 to 7 nights/week. This can decrease patients' optimal compliance. Nevertheless, over the recent decades, desferal has caused dramatic strides in survival of thalassemia patients have occurred (12).

Only a few of iron chelating agents have demonstrated an acceptable oral bioavailability. Deferiprone is an effective oral iron chelator that can reduce iron overload and improve myocardial siderosis and cardiac function (13). Its efficacy and especially cardiac iron load by deferiprone in perfusion-dependent patients have been shown in some studies (14, 15). The most serious side effect of deferiprone is agranulocytosis however, temporary gastrointestinal symptoms such as nausea, vomiting, and abdominal pain, and increased liver enzymes are the most common reported adverse effects (16, 17). So, during the first year of therapy, patients on deferiprone require a weekly complete blood count and then every two weeks to detect serious neutropenia/ agranulocytosis (18, 13). Deferasirox (Exjade^®^) was initially marketed by Novartis Company and then approved by the US FDA in November 2005. Deferasirox is an orally active tridentate chelator that selectively binds to the ferric (Fe3+) form and mobilizes iron stores with high selectivity to iron and low affinity for trace metals, such as zinc or copper (19, 20). This seems having similar efficacy compared to deferoxamine (21). Deferasirox is generally well-tolerated in adults, adolescents, and children above 2 years old. Single daily doses of 10 to 40 mg/kg deferasirox could reduce serum ferritin level, liver and cardiac iron concentration in adult and pediatric iron overload beta-thalassemia patients in many clinical studies (22-24).

Osveral^®^ (a brand name of deferasirox) is an Iranian product, was manufactured and supplied by Osvah Company. Osveral^®^ has been approved by the Food and Drug Administration of Iran. So far, no randomized clinical trial has been done to compare safety and efficacy of Osveral^®^ with Exjade^®^ in Iranian patients. Therefore, this study was proposed to compare the efficacy and safety between Osveral^®^ and Exjade^®^ in β-thalassemia major patients with transfusion induced iron overload.

## Methods

**Study design:** This randomized open-label clinical trial was performed on β-thalassemia major patients, who attended the thalassemia ward of Bu-Ali Sina Hospital in Sari, Iran. Ethical authorization has been approved with No. IR.MAZUMS.REC 94-1069 by the Ethics Committee of Mazandaran University of Medical Sciences and it was approved in the Iranian registry of clinical trial by registration code of IRCT20090813002342N9. The sample size estimation was performed based on the results of Eshghi et al.’s (25) study and considering 95% confidence level and 80% power of study that was calculated among 44 subjects for each group. Sampling of eligible patients performed from May 2015 to June 2016 and all patients or their parents signed the consent form. Prior to study enrolment, informed consent was obtained from each patient.**Eligibility criteria:** The criteria for screening and entering the study were male or female patients with β-thalassemia major, age>2 year, ferritin level above 1000 mg/dl or the volume of blood transfusion above 100 mL/kg. Exclusion criteria were pregnancy or breast-feeding state, progressive or persistent enhancement in the creatinine level, any heart, auditory or ophthalmic problems, hepatitis B (HBV) or C (HBC), and human immunodeficiency virus (HIV) infections. Also, patients with persistent liver transaminases more than 5 times of the normal level, severe nausea and vomiting, hypersensitivity to deferasirox, severe skin rashes, progressive proteinuria, non-compliant or unreliable patients were excluded.

**Treatment protocols:** Patients who met the inclusion criteria and completed informed consent assigned to enter two groups using simple randomization method (random number generation software). If it was an odd number, the patient was allocated in group 1 and if it was even number, the patient was assigned to group 2. The randomization process was performed by the ward supervisor (who was independent of the principal investigators) before a patient entered the study. All patients received a single daily dose of 30 mg/kg of either Exjade^®^ or Osveral^®^ 30 minutes before breakfast for 6 months. Primary outcome was the bimonthly changes in mean serum ferritin concentration during 6 months of treatment in comparison to baseline. The measurement of serum ferritin concentration is a comfortable, inexpensive and the most common method for evaluating total body iron. Secondary outcomes included mean changes of cardiac and liver MRI T2* between baseline and one year later, drug safety and tolerability. Mean serum ferritin levels, measured during the 1 month before the start of the treatment was considered as the baseline value and was compared with post-treatment values checked every two months. Measurement of T2* by magnetic resonance imaging (MRI) is a noninvasive way to assess iron levels in the heart, liver and endocrine organs. Hemosiderin molecules create local changes in the magnetic field; more iron load leads to increased magnetic field changes. This magnetic field change causes more rapid MRI signal decay rates. T2* signal decay rates are proportional to the iron levels in the tissue (26). We assessed the efficacy of the two drugs on improving cardiac and hepatic iron load as measured by MRI T2* imaging before and a year after starting of the treatments. For liver, values>6.3 ms were considered as normal, 2.8-6.3 ms as mild, 1.4-2.8 ms as moderate and <1.4 ms as severe iron overload. For heart, MRI T2* values >20 ms were considered as normal, 14-20 ms as mild, 10-14 ms as moderate and <10 ms as severe iron overload (27).

**Safety and Monitoring:** Laboratory tests and physical examination were performed prior to enrolment of patients in this study: complete blood count, serum creatinine, blood urea nitrogen, liver transaminases, serum ferritin levels and urine analysis (28). Bimonthly laboratory follow-up profiles were asked during the study. Serologic tests for HCV, HBV and HIV infection were performed at the beginning of the study to exclude affected or doubtful cases from the study. Echocardiography or MRI T2* (for patients>10 years old), and evaluation of auditory and ophthalmic system at the beginning of treatment were considered but not mandatory for all asymptomatic and clinically normal cases based on available facilities and the physician's judgment. If serum creatinine rises above 50% of baseline in two consecutive times, it was considered to be “raising creatinine”. More than 5-fold increase the normal serum transaminases level was considered as severe liver dysfunction (29).

Statistical analysis: Statistical analysis was done using the Statistics Program for Social Sciences for Windows (SPSS 20.0, SPSS Inc., Chicago, IL, USA). Quantitative data are presented as mean and standard deviation (SD). Independent sample t-test (or nonparametric Mann-Whitney U-test) and paired sample t-test were used to compare continuous variables (serum ferritin levels, cardiac and liver MRI T2* alteration, renal and liver function test) between two groups and intra group, respectively. Comparison of categorical variables (male/female ratio) was done with chi-square test and p-values less than 0.05 were defined as statistically significant.

## Results

**Figure 1:**
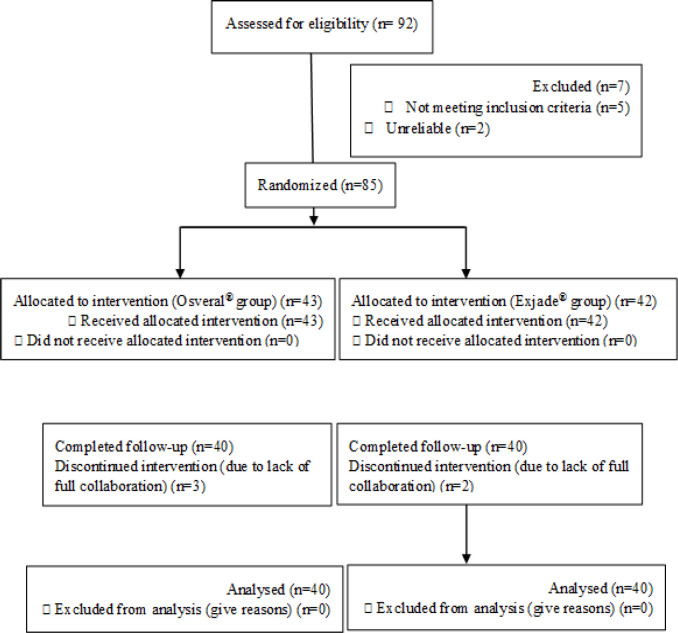
Screening, randomization and follow up of study subjects

Of the 92 patients evaluated to enter into the study, 7 of them were excluded and the remaining 85 subjects randomly were assigned to Osveral^®^ and Exjade^®^ groups. Five patients were excluded (three subjects in Osveral^®^ group and 2 in Exjade^®^ group) due to lack of collaboration during the study and finally 80 patients (40 in each group) were analyzed (figure 1). 

Baseline demographics and characteristics of patients in two groups were presented in table 1. There were no significant differences between two arms as regard age, weight, sex, mean baseline serum ferritin, transfused blood volume and interval of blood transfusion. Out of 40 cases male/female ratio in Osveral^®^ and Exjade^®^ groups were 21/19 and 19/21, respectively. The mean age of patients in Osveral^®^ group was 24.1±5.9 y (8-35) and in Exjade^®^ group was 22.5±6.5 y (8-34) years (P=0.27) that10% and 20% of them less than or equal to 18 years in the two groups, respectively.

All 80 patients were assessed for heart and liver iron overloading by MRI T2* and total body iron load using serum ferritin measurements. At baseline, there were no significant differences in serum ferritin levels between two study arms (p=0.31). After 2 months, the difference of mean serum ferritin between two study groups was significant (P=0.012), so that it decreased in Osveral^®^ group and increased slightly in Exjade^®^ group. At the end of fourth months, the difference of mean serum ferritin between two groups was borderline significantly (p=0.05), so Osveral^®^ decreased serum ferritin level faster than Exjade^®^ during the initial 4 months of the study but at the end of six months, we observed no significant difference in declined mean serum ferritin between two medicines (P= 0.1) (table 2). Both drugs were able to decrease the average serum ferritin meaningfully. The mean serum ferritin level significantly decreased from 1772.4±987.8 to 1252.1±746.7 in Osveral^®^ group (p<0.01) and from 2002.8±1044.6 to 1597.1±1068.8 in Exjade^®^ group (p<0.01).

For accurate analysis of the results of the study, we determined the mean changes of bimonthly serum ferritin during 6 months' treatment in comparison to baseline in Osveral^®^ and Exjade^®^ groups (table 3).

**Table 1:** Baseline demographics and characteristics of patients

Characteristics	Osveral^®^ Mean±SD	Exjade^®^ Mean ± SD	P value
**Age (year)**	24.1±5.9	22.5 ±6.5	0.27
**Weight (kg)**	52.5±11.3	48.7 ± 9.1	0.28
**Mean baseline serum ferritin (ng/mL)**	1772.4±987.8	2002.8 ± 1044.6	0.31
**Transfused blood volume in a month (mL)**	668.7**±**287.4	661.4 ± 367.2	0.92
**Interval of blood transfusion (day)**	27.6±3.9	29.6 **± **7.5	0.33

kg: kilogram; mL: milliliter; ng/mL: Nanograms per milliliter; SD: Standard Deviation

**Table 2:** Mean serum ferritin levels in two groups during 6 months' study (ng/mL)

	Osveral^®^ Mean ± SD	Exjade^®^ Mean ± SD	P value
**Baseline**	1772.4 ± 987.8	2002.8 ± 1044.6	0.31
**At the end of second month**	1480.2 ± 845.5	2064.1 ± 1169.0	0.012
**At the end of fourth month**	1547.6 ± 877.4	1977.2 ±1051.1	0.051
**At the end of sixth month**	1252.1 ± 1044.6	1597.1 ± 1086.8	0.10

SD: Standard Deviation

**Table 3:** Mean and SD serum ferritin changes relative to base value in two groups during the study (ng/mL)

	At the end of 2^th^month	P value	At the end of 4^th^ month	P value	At the end of 6^th^ month	P value
**Osveral** ^®^	-292.2 ± 603.9	<0.01	-224.8 ± 665.5	0.11	-520.4 ± 649.5	0.36
**Exjade** ^®^	61.2 ± 559.1		-25.6 ± 383.3		-405.7 ± 448.5	

Changes in heart and liver iron overload assessed by MRI T2* After a year, the mean cardiac MRI T2* in Osveral^®^ group patients were changed from 25.9±9.6 ms (baseline) to 25.4±9.7 ms and in Exjade^®^ group from 24.8±9.2 ms to 26.9±5.9 ms, with no significant difference between two groups (P=0.43). Mean liver MRI T2* for Osveral^®^ and Exjade^®^ groups were reported 8.6±6.4 ms (baseline 6.3±4.7) and 6.3±4 ms (baseline 4.9±3.5) after one year, respectively, with no significant difference observed between two study arms (P= 0.1) (table 4). Average liver MRI T2* alteration at the beginning and after one year were 2.3±4.5 and 1.4±2.8 ms in Osveral^®^ and Exjade^®^ groups, respectively, and there was no meaningful significant difference (P=0.6).

**Side effects:** The most common side effects included transient increased creatinine, 2 (5%) of cases in each group, elevated liver enzymes 1(2.5%) subject in Osveral^®^ and 2 subjects (5%) in Exjade^®^ group), and rash 2(5%) subjects in Osveral^®^ and 2 (2.5%) patients in Exjade^®^ group). Mean serum and urine creatinine, alanine aminotransferase (ALT) and aspartate aminotransferase (AST) as indicators for renal and liver functions in β-thalassemia patients receiving Osveral^®^ or Exjade^®^ were presented in table 5. There were no significant differences in the mean of biochemical parameters between two groups in the beginning, 2, 4 and 6 months after interventions.

**Table 4:** Cardiac and liver MRI T2* alteration in patients with beta thalassemia major receiving Exjade® or Osveral®

Variables		Osveral^®^ Mean ± SD	Exjade^®^ Mean ± SD	P value
Cardiac MRI T2* **(millisecond)**	baseline	25.9 ± 9.6	24.8 ± 9.2	0.63
	After a year	25.4 ± 9.7	26.9 ± 5.9	0.43
Liver MRI T2* **(millisecond)**	baseline	6.3 ± 4.7	4.9 ± 3.5	0.17
	After a year	8.6 ± 6.4	6.3 ± 4	0.1

MRI T2*: Magnetic Resonance Imaging T2*; SD: Standard Deviation

**Table 5:** Alterations in the renal and liver function during the study

		Osveral^®^ (Mean ± SD)	Exjade^®^ (Mean ± SD)	P value
**Creatinine (mg/dL)**	Baseline	0.78 **± **0.17	0.72 **± **0.12	0.1
	At the end of 2^th^ month	0.80 **± **0.18	0.74 **± **0.13	0.4
	At the end of 4^th^ month	0.83 **± **0.16	0.79 **± **0.14	0.9
	At the end of 6^th^ month	0.81 **± **0.15	0.76 **± **0.11	0.7
**Urinary protein ** **(mg/d)**	Baseline	15.6 **± **22.6	17.8 **± **25.9	0.6
	At the end of 2^th^ month	17.6 **± **35.1	19.2 **± **38.9	0.9
	At the end of 4^th^ month	10.7 **± **16.2	11.9 **± **7.3	0.7
	At the end of 6^th^ month	8.3 **± **13.4	10.5 **± **22.3	0.3
**Urinary creatinine** **(mg/dL)**	Baseline	121.7 **± **124.2	159.2 **± **221.7	0.5
	At the end of 2^th^ month	123.9 ± 91.3	148.7 **± **239.1	0.2
	At the end of 4^th^ month	83.5 **± **43.2	119.6 **± **221.2	0.4
	At the end of 6^th^ month	95.5 **± **35.2	113.9 **± **147.3	0.5
**Alanine aminotransferase** **(U/L)**	Baseline	24.7 ± 11.7	25.3 ± 12.8	0.84
	At the end of 2^th^ month	25.4 ± 13.6	25.2 ± 16.7	0.97
	At the end of 4^th^ month	23.4 ± 14.7	23.1 ± 10.4	0.89
	At the end of 6^th^ month	24.7 ± 13.1	20.5 ± 10.2	0.13
**Aspartate** **Aminotransferase** **(U/L)**	Baseline	26.2 ± 18.3	21.1 ± 19.2	0.25
	At the end of 2^th^ month	28.5 ± 24.0	27.9±22.1	0.91
	At the end of 4^th^ month	22.9 ± 19.3	23.4 ± 14.0	0.9
	At the end of 6^th^ month	24.5 ± 15.1	19.8 ± 13.4	0.15

mg/dL: milligrams per deciliter; mg/d: Milligrams per day; SD: Standard Deviation; U/L: Units per litre

## Discussion

To our knowledge, this study is the first randomized clinical trial that compares the efficacy and safety of Osveral^®^ (the Iranian brand name of deferasirox) versus Exjade^®^ (Novartis Pharmaceuticals Corporation) in the treatment of transfusional iron overload in Iranian patients with beta-thalassemia major. The primary outcome was the mean of bimonthly changes in serum ferritin levels during 6 months of the treatment in comparison to baseline. Secondary outcomes were safety, tolerability during the study and mean changes of cardiac and liver MRI T2* after a year. The mean serum ferritin level in Exjade^®^ group at the beginning of the study was 2002.8±844.6 ng/mL and six months after receiving Exjade^®^ changed to 1597.1±786.8 (p<0.05) that was in contrast to results of Alavi et al.’s study. They have reported the efficacy and safety of deferasirox (Exjade^®^) in the treatment of iron-overloaded in Iranian β-thalassemia major patients. Mean serum ferritin level in patients (n=30), before the study was 1892.5±185.9 ng/mL and after receiving Exjade^®^ was 1762±206.8 ng/mL (P=0.25) (30). 

In our study, serum ferritin levels significantly declined from second to sixth month in Osveral^®^ group (from 1772.4±987.8 to1252.1±1044.6ng/mL, p=0.01) that was similar to results of Molavi et al.’s study. They evaluated the effectiveness and safety of Osveral^®^ on 80 major thalassemia patients with iron overload. In the mentioned study, serum ferritin levels significantly decreased from first month to one year after (p<0.05). However, small increases were observed during the third and fourth months of the treatment in contrast to our results that serum ferritin decreased constantly along the study (31). 

In the present study, the mean changes of serum ferritin after 6 months in comparison to baseline in Osveral^®^ group was -520.3±241.1 ng/mL (-29.3%) and significant decrease was seen in serum ferritin during 6 months of receiving Osveral^®^ (P= 0.01). Our results were the same as the report of Eshghi et al.’s in decreasing of serum ferritin in Osveral^®^ group. This research group conducted a one-year prospective multicenter study on 407 Iranian major thalassemia patients receiving Osveral^®^. In comparison to baseline, the mean of relative changes of serum ferritin was -11.4% and significant decrease was seen in serum ferritin during one year of receiving Osveral^®^ (p<0.001) (25). In ESCALATOR study, the median serum ferritin decreased by 341 ng⁄mL in 52 weeks (22). In a non-randomized controlled trial by Ashayeri et al, the efficacy of deferasirox (Exjade^®^) versus Osveral^®^ was evaluated in the treatment of iron overload in Iranian β-thalassemia major patients. No statistically significant difference was seen in serum ferritin level after treatment between the two groups (32). In Jaiswal et al.’s study, the mean serum ferritin before deferasirox therapy with mean dose of 38 mg/kg/day was 3727.0 ng/mL and after 12 months, the mean decrease in serum ferritin was 1207.1 ng/mL (drop by 32.38%, p<0.001) (33). Cappellini et al. found median decrease in serum ferritin from 2117 to 1124 after using deferasirox ≥4 y (p<0.001) (29). A statistically significant median reduction in serum ferritin of 517 ng ⁄mL (p<0.001) was observed by Taher et al., and at the end of the study in patients whose therapeutic goal was reduction (baseline liver iron concentration (LIC): 7 mg Fe⁄g dw, n=215) (22). 

In the study of Merchant et al.,the mean baseline serum ferritin was 3859.8 ± 1690.70 ng/mL (1066-6725 ng/mL) and after 12 to 18 months of treatment with a mean dose of 33 mg/kg/day, the mean serum ferritin reduced to 2693.4±1831.5 ng/mL (drop by 30.2%, p<0.001) (34). YR Lai et al., reported that in Chinese patients using 33.6 mg/kg/day, the median ferritin reduction of -756 ng/mL (P=0.039) (35). A reduction in the serum ferritin levels was shown by Reddy Y et al. in 75% of patients (that responded to the treatment) from 4362.5 ng/mL (range 1337.8-17217 ng/mL) to 1869.25 ng/mL (range 634.4-3486.1 ng/mL) during deferasirox therapy (36).

In the current study, the mean cardiac MRI T2* changes inpatients using Osveral^®^ was similar to those receiving Exjade^®^. There were no significant differences between baseline and end values for intra-group and between two groups (P=0.43). Also no significant differences were observed between averages of liver MRI T2* in Osveral^®^ and Exjade^®^ groups (p=0.1). After a year, the means of liver MRI T2* alteration from the beginning were 2.3±4.5 and 1.4±2.8 ms in Osveral^®^ and Exjade^®^ groups, respectively, with no meaningful significant difference (P=0.6). These results were similar to Ashayeri et al. ‘s results that reported no significant difference after a year, regarding mean cardiac and liver MRI T2* between Osveral^®^ and Exjade^®^ groups (p=0.43 and 0.1 respectively) (32). Alavi et al. showed liver MRI T2* value after receiving Exjade^®^ improved significantly (P=0.002). Cardiac MRI T2∗ improved after the period of study but not significantly (P=0.2). Exjade^®^ was successful in 73.3% and 80% of patients in terms of improvement in the level of severity or maintenance of normal values of liver and cardiac iron concentration, respectively (30). Ashayeri et al. reported that reducing liver iron overload in Osveral^®^ group was statistically significant (P=0.007), while, in Exjade^®^ group, decreasing in cardiac iron overload was significant (p<0.001) (32).In Merchant et al.’s study, mean cardiac MRI T2* changed from 23.8±15.2 ms (6.24-69.2 ms) to 24.2±12.9 ms (increase of 1.6 %, p=0.87) after 12 to 18 months of deferasirox therapy on a mean dose of 33 mg/kg/day (34).

The most common adverse events of Osveral^®^ in our study were transient increase in serum creatinine (5%), elevation liver transaminases levels (2.5%) and skin rash (5%). Similarly, Molavi et al. reported increase of serum creatinine (5%) and liver enzymes (3.75%) were the most common adverse effects of Osveral^®^ (31). Also, Eshghi et al .showed that the most prevalent adverse effect of Osveral^®^ were temporary increase in serum creatinine (24.1%) and >5 time increase in transaminases level (5.89%) (25), while they were 5% and 2.5% of the participants in our study, respectively. Different definitions of transient increase of serum creatinine and serum liver transaminases might be associated to diversity in results. Skin rash and increasing liver transaminases levels in 16.7% and rising of serum creatinine in 26.7% were reported by Ashayeri et al. in patients that received Osveral^®^ (32).The most common side effects of Exjade^®^ in the present study were temporary increase in serum creatinine (5%), increase liver transaminases levels (5%) and skin rash (2.5%). Alavi et al. have reported increase in serum creatinine (26.7%), increased ALT and skin rash (16.7%) and as the most common adverse events of Exjade^®^ in Iranian patients (30). Perhaps the long time of their trial (18-month) in comparison to our study was responsible for this higher frequency of adverse effects. The other reason for the low rate of adverse events compared to other studies could be related to mild side effects which were not reported by patients. Ashayeri et al. reported skin rash, increasing liver transaminase levels and rising of serum creatinine in 17.9, 12.8 and 53.8%in Exjade^®^ group, respectively (32). 

Cappellini et al. have reported an increase in serum creatinine (38%) and skin rash (10.8%) in patients receiving Exjade^®^ (37). Nausea and vomiting (16.1%), skin rash (8%), increased ALT (5.5%) and an increase in serum creatinine (3.8%) have been found by Taher et al. as the most common adverse events of Exjade^®^ (38). Galanello et al. have also evaluated Exjade^®^ safety in a phase II clinical trial in 39 pediatric patients and have reported mild nausea in two adolescents and moderate skin rash in two children (39). The results of Hajigholami et al. showed that Osveral^®^ was as effective as desferal^®^ in reduction of serum ferritin levels and recommended an appropriate substitution for desferal in thalassemia patients (40).

In our opinion, no RCT has examined the efficacy of Exjade^®^ vs. Osveral^®^ in major β-thalassemia patients and the purpose of this study was to compare the efficacy of these drugs for decreasing of serum ferritin and iron removal of the liver and heart by MRI T2* value.

Our study results showed Osveral^®^ (Osvah Pharmaceutical Company, Iran) is as efficient as Exjade^®^ (Novartis Company, Switzerland) for the treatment of iron overload in Iranian patients with major β-thalassemia. Osveral^®^ could significantly decrease serum ferritin and improve heart and liver iron overload equal to Exjade^®^ during the study. Osveral^®^ can be a suitable cost-effective and a good alternative for Exjade^®^ in Iranian patients with major β-thalassemia.

## Funding:

This article was based on the doctoral thesis of Pharmacy of Bita Lashto-Aghaee and was financially supported by the Deputy of Research and Technology of Mazandaran University of Medical Sciences. The funders had no role in the design of the study, collection, analyses, or interpretation of data; in the writing of the manuscript, and in the decision to publish the results.

## Conflicts of Interest:

The authors declare that they have no conflicts of interest.
